# (5-Hy­droxy-3-methyl-5-phenyl-4,5-dihydro-1*H*-pyrazol-1-yl)(pyridin-4-yl)methanone monohydrate

**DOI:** 10.1107/S160053681005275X

**Published:** 2010-12-24

**Authors:** Hadi Kargar, Reza Kia, Fatemeh Froozandeh, Moayad Hossaini Sadr, Muhammad Nawaz Tahir

**Affiliations:** a Department of Chemistry, School of Science, Payame Noor University (PNU), Ardakan, Yazd, Iran; b Department of Chemistry, Science and Research Branch, Islamic Azad University, Tehran, Iran; c X-ray Crystallography Lab., Plasma Physics Research Center, Science and Research Branch, Islamic Azad University, Tehran, Iran; d Department of Chemistry, Azarbaijan University of Tarbiat Moallem, Tabriz, Iran; e Department of Physics, University of Sargodha, Punjab, Pakistan

## Abstract

In the title compound, C_16_H_15_N_3_O_2_·H_2_O, the mean plane of the approximately planar pyrazole ring [maximum deviation = 0.0474 (18) Å] makes dihedral angles of 86.32 (11) and 45.04 (10)° with the phenyl and pyridine rings, respectively. The dihedral angle between the phenyl and pyridine rings is 69.62 (11)°. In the crystal, inter­molecular O—H⋯O and O—H⋯N hydrogen bonds connect the components into chains along [010]. The crystal structure is further stabilized by π–π stacking inter­actions with centroid–centroid distances of 3.7730 (12) Å.

## Related literature

For standard values of bond lengths, see: Allen *et al.* (1987 ▶).
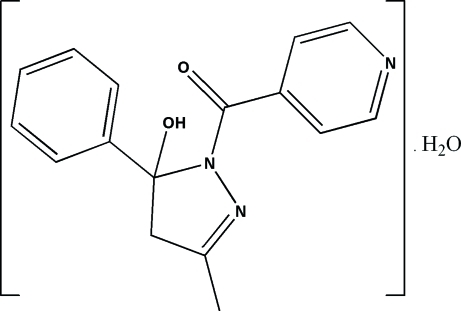


## Experimental

### 

#### Crystal data

C_16_H_15_N_3_O_2_·H_2_O
*M* *_r_* = 299.33
Monoclinic, 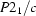

*a* = 16.9676 (10) Å
*b* = 7.0266 (5) Å
*c* = 12.6135 (6) Å
β = 93.004 (3)°
*V* = 1501.77 (16) Å^3^
*Z* = 4
Mo *K*α radiation
μ = 0.09 mm^−1^
*T* = 296 K
0.32 × 0.24 × 0.18 mm

#### Data collection

Bruker SMART APEXII CCD area-detector diffractometer
Absorption correction: multi-scan (*SADABS*; Bruker, 2005 ▶) *T*_min_ = 0.971, *T*_max_ = 0.983
8525 measured reflections
3059 independent reflections
1916 reflections with *I* > 2σ(*I*)
*R*_int_ = 0.038

#### Refinement

*R*[*F* ^2^ > 2σ(*F* ^2^)] = 0.048
*wR*(*F* ^2^) = 0.121
*S* = 1.03
3059 reflections
200 parameters
H-atom parameters constrained
Δρ_max_ = 0.15 e Å^−3^
Δρ_min_ = −0.18 e Å^−3^

### 

Data collection: *APEX2* (Bruker, 2005 ▶); cell refinement: *SAINT* (Bruker, 2005 ▶); data reduction: *SAINT*; program(s) used to solve structure: *SHELXTL* (Sheldrick, 2008 ▶); program(s) used to refine structure: *SHELXTL*; molecular graphics: *SHELXTL* and *PLATON* (Spek, 2009 ▶); software used to prepare material for publication: *SHELXTL* and *PLATON*.

## Supplementary Material

Crystal structure: contains datablocks global, I. DOI: 10.1107/S160053681005275X/lh5187sup1.cif

Structure factors: contains datablocks I. DOI: 10.1107/S160053681005275X/lh5187Isup2.hkl

Additional supplementary materials:  crystallographic information; 3D view; checkCIF report

## Figures and Tables

**Table 1:** Hydrogen-bond geometry (Å, °)

*D*—H⋯*A*	*D*—H	H⋯*A*	*D*⋯*A*	*D*—H⋯*A*
O1—H1⋯O1*W*^i^	0.92	1.84	2.7490 (19)	173
O1*W*—H1*W*1⋯O2^ii^	0.92	1.89	2.8003 (19)	169
O1*W*—H2*W*1⋯N3^iii^	0.85	2.13	2.976 (2)	173

Symmetry codes: (i) 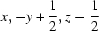; (ii) 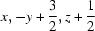; (iii) 
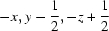
.

**Fig. 1.**
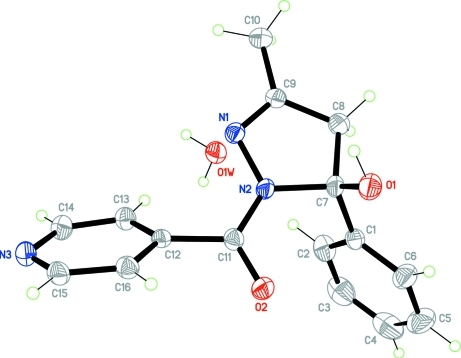
The asymmetric unit of the title compound, showing 30% probability displacement ellipsoids and the atomic numbering.

**Fig. 2.**
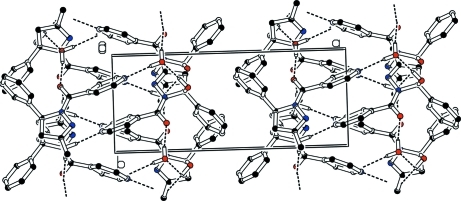
The packing of the compound viewed along the c-axis showing 1-D extended chains along the b-axis through hydrogen bonds. All H atoms removed except those involved in the hydrogen bonds. Hydrogen bonds are shown as dashed lines.

## supplementary crystallographic information

### Comment

The asymmetric unit of the title compound, Fig. 1, comprises a substituted pyrazole molecule and a solvent water molecule. The bond lengths (Allen *et al.,* 1987) and angles are within the normal ranges. The dihedral angle the mean plane of the pyrazole ring makes with the phenyl and pyridine rings are 86.32 (11) and 45.04 (10)°, respectively. The dihedral angle between the phenyl ring and the pyridine ring is 69.62 (11)Å.

In the crystal structure, intermolecular O—H···O and O—H···N hydrogen bonds connect the components of the structure to form one-dimensional chains along [0 1 0]. The crystal structure is further stabilized by intermolecular π–π stacking interactions [*Cg*1···*Cg*1^iv^ = 3.7730 (11)Å, (iv) -*x*, 1 - *y*, -*z*, *Cg*1 is the centroid of the C12-C16/N3 ring].

### Experimental

The title compound was synthesized by adding isoniazide (2 mmol) to a solution of benzoylacetone (2 mmol) in ethanol (20 ml). The mixture was refluxed with stirring for half an hour. The resultant solution was filtered and the white single crystals suitable for *X*-ray structure determination were recrystallized from ethanol by slow evaporation of the solvents at room temperature over several days.

### Refinement

The H atoms of the water and hydroxy groups were located in a difference Fourier map and constraied to refine on the parent atom with U_iso_(H) = 1.5 U_eq_(O), see Table 1. The remaining H atoms were positioned geometrically with C—H = 0.93-0.97 Å and included in a riding-model approximation with U_iso_(H) = 1.2 or 1.5 U_eq_(C). A rotating group model was used for the methyl group.

### Crystal data

**Table:**

C_16_H_15_N_3_O_2_·H_2_O	*F*(000) = 632
*M**_r_* = 299.33	*D*_x_ = 1.324 Mg m^−^^3^
Monoclinic, *P*2_1_/*c*	Mo *K*α radiation, λ = 0.71073 Å
Hall symbol: -P 2ybc	Cell parameters from 2540 reflections
*a* = 16.9676 (10) Å	θ = 2.5–27.5°
*b* = 7.0266 (5) Å	µ = 0.09 mm^−^^1^
*c* = 12.6135 (6) Å	*T* = 296 K
β = 93.004 (3)°	Prism, white
*V* = 1501.77 (16) Å^3^	0.32 × 0.24 × 0.18 mm
*Z* = 4	

### Data collection

**Table:**

Bruker SMART APEXII CCD area-detector diffractometer	3059 independent reflections
Radiation source: fine-focus sealed tube	1916 reflections with *I* > 2σ(*I*)
graphite	*R*_int_ = 0.038
φ and ω scans	θ_max_ = 26.5°, θ_min_ = 2.4°
Absorption correction: multi-scan (*SADABS*; Bruker, 2005)	*h* = −18→21
*T*_min_ = 0.971, *T*_max_ = 0.983	*k* = −7→8
8525 measured reflections	*l* = −15→14

### Refinement

**Table:**

Refinement on *F*^2^	Primary atom site location: structure-invariant direct methods
Least-squares matrix: full	Secondary atom site location: difference Fourier map
*R*[*F*^2^ > 2σ(*F*^2^)] = 0.048	Hydrogen site location: inferred from neighbouring sites
*wR*(*F*^2^) = 0.121	H-atom parameters constrained
*S* = 1.03	*w* = 1/[σ^2^(*F*_o_^2^) + (0.0554*P*)^2^] where *P* = (*F*_o_^2^ + 2*F*_c_^2^)/3
3059 reflections	(Δ/σ)_max_ < 0.001
200 parameters	Δρ_max_ = 0.15 e Å^−^^3^
0 restraints	Δρ_min_ = −0.18 e Å^−^^3^

### Special details

**Table:**

Geometry. All esds (except the esd in the dihedral angle between two l.s. planes) are estimated using the full covariance matrix. The cell esds are taken into account individually in the estimation of esds in distances, angles and torsion angles; correlations between esds in cell parameters are only used when they are defined by crystal symmetry. An approximate (isotropic) treatment of cell esds is used for estimating esds involving l.s. planes.
Refinement. Refinement of F^2^ against ALL reflections. The weighted R-factor wR and goodness of fit S are based on F^2^, conventional R-factors R are based on F, with F set to zero for negative F^2^. The threshold expression of F^2^ > 2sigma(F^2^) is used only for calculating R-factors(gt) etc. and is not relevant to the choice of reflections for refinement. R-factors based on F^2^ are statistically about twice as large as those based on F, and R- factors based on ALL data will be even larger.

### Fractional atomic coordinates and isotropic or equivalent isotropic displacement parameters (Å^2^)

**Table:**

	*x*	*y*	*z*	*U*_iso_*/*U*_eq_	
O1	0.32045 (8)	0.3424 (2)	−0.01490 (10)	0.0461 (4)	
H1	0.2849	0.2478	−0.0322	0.069*	
O2	0.22503 (8)	0.70361 (19)	0.00147 (10)	0.0455 (4)	
O1W	0.21323 (8)	0.4293 (2)	0.41563 (10)	0.0522 (4)	
H1W1	0.2123	0.5449	0.4498	0.078*	
H2W1	0.1656	0.3911	0.4143	0.078*	
N1	0.17899 (8)	0.3038 (2)	0.15669 (11)	0.0339 (4)	
N2	0.22186 (8)	0.4400 (2)	0.10150 (11)	0.0347 (4)	
N3	−0.05005 (9)	0.7733 (2)	0.10396 (13)	0.0431 (4)	
C1	0.36077 (10)	0.5444 (3)	0.12956 (14)	0.0370 (5)	
C2	0.35148 (13)	0.6223 (3)	0.22873 (16)	0.0544 (6)	
H2	0.3115	0.5788	0.2703	0.065*	
C3	0.40176 (17)	0.7653 (4)	0.2661 (2)	0.0726 (8)	
H3A	0.3947	0.8188	0.3323	0.087*	
C4	0.46131 (16)	0.8286 (4)	0.2071 (3)	0.0770 (8)	
H4	0.4951	0.9238	0.2333	0.092*	
C5	0.47118 (14)	0.7520 (4)	0.1099 (2)	0.0726 (8)	
H5	0.5119	0.7947	0.0694	0.087*	
C6	0.42080 (12)	0.6104 (3)	0.07061 (17)	0.0521 (6)	
H6	0.4277	0.5596	0.0036	0.062*	
C7	0.30659 (10)	0.3854 (3)	0.09099 (13)	0.0359 (5)	
C8	0.31075 (11)	0.2087 (3)	0.16286 (16)	0.0449 (5)	
H8A	0.3310	0.1000	0.1255	0.054*	
H8B	0.3443	0.2320	0.2262	0.054*	
C9	0.22789 (11)	0.1762 (3)	0.19037 (13)	0.0339 (4)	
C10	0.20331 (12)	0.0106 (3)	0.25333 (15)	0.0469 (5)	
H10A	0.1476	0.0176	0.2628	0.070*	
H10B	0.2314	0.0116	0.3214	0.070*	
H10C	0.2150	−0.1048	0.2166	0.070*	
C11	0.18790 (11)	0.5957 (3)	0.05705 (13)	0.0334 (4)	
C12	0.10340 (10)	0.6415 (2)	0.07655 (13)	0.0297 (4)	
C13	0.07060 (11)	0.6357 (3)	0.17484 (14)	0.0375 (5)	
H13	0.0990	0.5858	0.2335	0.045*	
C14	−0.00432 (12)	0.7045 (3)	0.18447 (15)	0.0430 (5)	
H14	−0.0247	0.7031	0.2515	0.052*	
C15	−0.01844 (11)	0.7738 (3)	0.00927 (15)	0.0407 (5)	
H15	−0.0493	0.8176	−0.0488	0.049*	
C16	0.05681 (11)	0.7135 (3)	−0.00760 (14)	0.0361 (5)	
H16	0.0764	0.7210	−0.0750	0.043*	

### Atomic displacement parameters (Å^2^)

**Table:**

	*U*^11^	*U*^22^	*U*^33^	*U*^12^	*U*^13^	*U*^23^
O1	0.0399 (8)	0.0500 (9)	0.0490 (8)	−0.0025 (7)	0.0074 (6)	−0.0062 (7)
O2	0.0370 (8)	0.0445 (9)	0.0556 (8)	0.0018 (7)	0.0082 (6)	0.0166 (7)
O1W	0.0428 (8)	0.0447 (9)	0.0690 (9)	−0.0001 (7)	0.0023 (6)	−0.0063 (7)
N1	0.0297 (9)	0.0353 (9)	0.0368 (8)	−0.0024 (8)	0.0008 (7)	0.0024 (7)
N2	0.0240 (8)	0.0362 (9)	0.0438 (8)	0.0016 (7)	0.0022 (6)	0.0106 (7)
N3	0.0319 (9)	0.0421 (10)	0.0553 (11)	0.0033 (8)	0.0027 (8)	−0.0013 (8)
C1	0.0274 (10)	0.0373 (11)	0.0457 (11)	0.0028 (9)	−0.0031 (8)	0.0043 (9)
C2	0.0458 (13)	0.0606 (16)	0.0564 (13)	0.0085 (12)	−0.0017 (10)	−0.0066 (11)
C3	0.0676 (18)	0.0689 (19)	0.0786 (17)	0.0129 (16)	−0.0232 (14)	−0.0247 (14)
C4	0.0537 (17)	0.0506 (17)	0.123 (2)	−0.0011 (14)	−0.0303 (16)	−0.0094 (16)
C5	0.0484 (15)	0.0663 (19)	0.102 (2)	−0.0183 (14)	−0.0027 (14)	0.0144 (16)
C6	0.0413 (12)	0.0551 (15)	0.0598 (13)	−0.0080 (11)	0.0028 (10)	0.0046 (11)
C7	0.0253 (10)	0.0393 (12)	0.0434 (11)	0.0041 (9)	0.0048 (8)	0.0036 (9)
C8	0.0352 (11)	0.0387 (12)	0.0605 (12)	0.0049 (10)	0.0006 (9)	0.0118 (10)
C9	0.0355 (11)	0.0314 (11)	0.0345 (9)	0.0005 (9)	0.0000 (8)	−0.0005 (8)
C10	0.0472 (12)	0.0370 (12)	0.0567 (12)	0.0009 (11)	0.0055 (9)	0.0069 (10)
C11	0.0294 (10)	0.0355 (11)	0.0349 (10)	0.0017 (9)	−0.0009 (8)	0.0008 (9)
C12	0.0289 (10)	0.0249 (10)	0.0351 (10)	−0.0014 (8)	0.0004 (8)	−0.0009 (8)
C13	0.0348 (11)	0.0420 (12)	0.0355 (10)	0.0022 (10)	−0.0015 (8)	0.0012 (9)
C14	0.0381 (12)	0.0480 (13)	0.0431 (11)	−0.0005 (10)	0.0053 (9)	−0.0035 (9)
C15	0.0343 (11)	0.0386 (12)	0.0480 (12)	0.0020 (10)	−0.0095 (9)	0.0048 (9)
C16	0.0363 (11)	0.0358 (11)	0.0360 (10)	0.0017 (9)	−0.0013 (8)	0.0013 (8)

### Geometric parameters (Å, °)

**Table:**

O1—C7	1.401 (2)	C5—C6	1.386 (3)
O1—H1	0.9166	C5—H5	0.9300
O2—C11	1.229 (2)	C6—H6	0.9300
O1W—H1W1	0.9200	C7—C8	1.536 (3)
O1W—H2W1	0.8517	C8—C9	1.483 (3)
N1—C9	1.279 (2)	C8—H8A	0.9700
N1—N2	1.408 (2)	C8—H8B	0.9700
N2—C11	1.345 (2)	C9—C10	1.481 (3)
N2—C7	1.501 (2)	C10—H10A	0.9600
N3—C15	1.334 (2)	C10—H10B	0.9600
N3—C14	1.336 (2)	C10—H10C	0.9600
C1—C6	1.373 (3)	C11—C12	1.502 (2)
C1—C2	1.382 (3)	C12—C16	1.386 (2)
C1—C7	1.511 (3)	C12—C13	1.386 (2)
C2—C3	1.384 (3)	C13—C14	1.371 (3)
C2—H2	0.9300	C13—H13	0.9300
C3—C4	1.360 (4)	C14—H14	0.9300
C3—H3A	0.9300	C15—C16	1.372 (3)
C4—C5	1.358 (4)	C15—H15	0.9300
C4—H4	0.9300	C16—H16	0.9300
			
C7—O1—H1	104.0	C9—C8—H8A	110.9
H1W1—O1W—H2W1	104.4	C7—C8—H8A	110.9
C9—N1—N2	107.33 (14)	C9—C8—H8B	110.9
C11—N2—N1	122.54 (15)	C7—C8—H8B	110.9
C11—N2—C7	124.25 (15)	H8A—C8—H8B	108.9
N1—N2—C7	113.06 (13)	N1—C9—C10	122.21 (17)
C15—N3—C14	115.90 (17)	N1—C9—C8	114.87 (16)
C6—C1—C2	118.6 (2)	C10—C9—C8	122.91 (17)
C6—C1—C7	122.12 (17)	C9—C10—H10A	109.5
C2—C1—C7	119.22 (17)	C9—C10—H10B	109.5
C1—C2—C3	119.9 (2)	H10A—C10—H10B	109.5
C1—C2—H2	120.1	C9—C10—H10C	109.5
C3—C2—H2	120.1	H10A—C10—H10C	109.5
C4—C3—C2	120.9 (2)	H10B—C10—H10C	109.5
C4—C3—H3A	119.5	O2—C11—N2	121.24 (17)
C2—C3—H3A	119.5	O2—C11—C12	118.90 (16)
C5—C4—C3	119.6 (2)	N2—C11—C12	119.85 (16)
C5—C4—H4	120.2	C16—C12—C13	117.15 (17)
C3—C4—H4	120.2	C16—C12—C11	117.65 (15)
C4—C5—C6	120.3 (2)	C13—C12—C11	124.86 (16)
C4—C5—H5	119.9	C14—C13—C12	119.10 (17)
C6—C5—H5	119.9	C14—C13—H13	120.4
C1—C6—C5	120.7 (2)	C12—C13—H13	120.4
C1—C6—H6	119.7	N3—C14—C13	124.36 (18)
C5—C6—H6	119.7	N3—C14—H14	117.8
O1—C7—N2	110.42 (13)	C13—C14—H14	117.8
O1—C7—C1	109.67 (14)	N3—C15—C16	123.97 (17)
N2—C7—C1	110.60 (15)	N3—C15—H15	118.0
O1—C7—C8	112.56 (16)	C16—C15—H15	118.0
N2—C7—C8	99.74 (14)	C15—C16—C12	119.47 (17)
C1—C7—C8	113.51 (15)	C15—C16—H16	120.3
C9—C8—C7	104.34 (15)	C12—C16—H16	120.3
			
C9—N1—N2—C11	−179.02 (16)	N2—C7—C8—C9	7.23 (18)
C9—N1—N2—C7	5.31 (18)	C1—C7—C8—C9	124.87 (16)
C6—C1—C2—C3	0.6 (3)	N2—N1—C9—C10	179.31 (16)
C7—C1—C2—C3	178.63 (19)	N2—N1—C9—C8	0.2 (2)
C1—C2—C3—C4	−1.1 (4)	C7—C8—C9—N1	−5.2 (2)
C2—C3—C4—C5	0.7 (4)	C7—C8—C9—C10	175.69 (17)
C3—C4—C5—C6	0.2 (4)	N1—N2—C11—O2	−173.53 (15)
C2—C1—C6—C5	0.2 (3)	C7—N2—C11—O2	1.7 (3)
C7—C1—C6—C5	−177.7 (2)	N1—N2—C11—C12	7.4 (2)
C4—C5—C6—C1	−0.6 (4)	C7—N2—C11—C12	−177.38 (15)
C11—N2—C7—O1	−64.9 (2)	O2—C11—C12—C16	39.6 (2)
N1—N2—C7—O1	110.71 (15)	N2—C11—C12—C16	−141.39 (18)
C11—N2—C7—C1	56.7 (2)	O2—C11—C12—C13	−133.48 (19)
N1—N2—C7—C1	−127.72 (15)	N2—C11—C12—C13	45.6 (3)
C11—N2—C7—C8	176.49 (17)	C16—C12—C13—C14	−1.5 (3)
N1—N2—C7—C8	−7.92 (18)	C11—C12—C13—C14	171.52 (18)
C6—C1—C7—O1	−8.6 (2)	C15—N3—C14—C13	−0.4 (3)
C2—C1—C7—O1	173.52 (17)	C12—C13—C14—N3	2.0 (3)
C6—C1—C7—N2	−130.57 (19)	C14—N3—C15—C16	−1.7 (3)
C2—C1—C7—N2	51.5 (2)	N3—C15—C16—C12	2.1 (3)
C6—C1—C7—C8	118.3 (2)	C13—C12—C16—C15	−0.4 (3)
C2—C1—C7—C8	−59.6 (2)	C11—C12—C16—C15	−173.97 (17)
O1—C7—C8—C9	−109.82 (17)		

### Hydrogen-bond geometry (Å, °)

**Table:**

*D*—H···*A*	*D*—H	H···*A*	*D*···*A*	*D*—H···*A*
O1—H1···O1W^i^	0.92	1.84	2.7490 (19)	173.
O1W—H1W1···O2^ii^	0.92	1.89	2.8003 (19)	169.
O1W—H2W1···N3^iii^	0.85	2.13	2.976 (2)	173.

Symmetry codes: (i) *x*, −*y*+1/2, *z*−1/2; (ii) *x*, −*y*+3/2, *z*+1/2; (iii) −*x*, *y*−1/2, −*z*+1/2.
